# Design Suggestions on Resistance from Flange of Sorbite Stainless Steel Plate Girder under Shear

**DOI:** 10.3390/ma15228069

**Published:** 2022-11-15

**Authors:** Xuanyi Xue, Neng Wang, Lepeng Huang, Jianmin Hua, Fei Wang, Zengshun Chen, Ji Liao, Letian Hai

**Affiliations:** 1 School of Civil Engineering, Chongqing University, Chongqing 400045, China; 2 Key Laboratory of New Technology for Construction of Cities in Mountain Area, Chongqing University, Ministry of Education, Chongqing 400045, China; 3 School of Management Science and Real Estate, Chongqing University, Chongqing 400045, China; 4 The Third Construction Limited Company of China Construction Third Engineering Bureau, Wuhan 430070, China; 5 Key Laboratory of Civil Engineering Safety and Durability of China Education Ministry, Department of Civil Engineering, Tsinghua University, Beijing 100084, China

**Keywords:** sorbite stainless steel, material property, ultimate resistance, flange, design equation, parametric study

## Abstract

A new S600E sorbite stainless steel (SS), which performs outstanding mechanical properties, was introduced in a plate girder to enhance the resistant performance and durability. The resistance from the flange for S600E sorbite SS plate girders developing post-buckling capacity was investigated through numerical analyses, which included the material and geometrical nonlinearity. The value of distance between plastic hinges performed significant effects on resistance from flange. There was a certain distribution range of the flange plastic hinge. Hence, it was difficult to determine the value of distance between plastic hinges accurately based merely on the failure behavior. Considering the theoretical basis of EN 1993-1-4: 2006+A1, the new methods to obtain resistance from the flange and determine the value of distance between the plastic hinges were proposed to avoid the aforementioned error. The parametric study was conducted to investigate the effect of key parameters on the resistance from the flange. To take the above effect into account, a correction factor was proposed for the design equation in EN 1993-1-4: 2006+A1 to predict the distance between flange plastic hinges accurately. The comparison was conducted to validate the accuracy of the proposed equations. The results indicated that the new modified equation could be used to predict the resistance from the flange of the S600E sorbite SS plate girder more accurately.

## 1. Introductions

**Figure 1:**
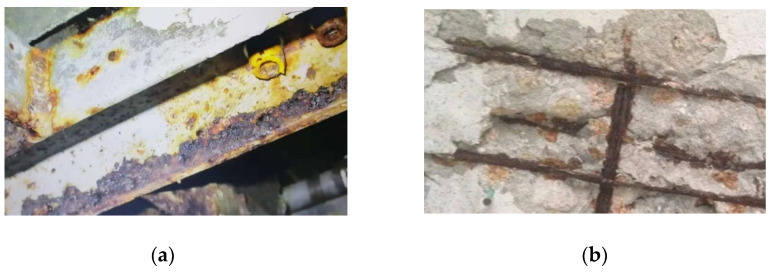
Corrosion in structural and reinforcing steels. (**a**) structural steel; (**b**) reinforcing steel.

Corrosion performed significant adverse effects on the durability of structures [1,2,3] (Figure 1). When structures were exposed to a corrosive environment, corrosion reduced the effective cross-section of structural and reinforcing steel obviously [4,5,6,7,8,9]. The durability of reinforced concrete structures and steel structures was reduced significantly [10,11]. Therefore, the stainless steel was widely used in structures to solve the above issue [12,13,14,15,16,17]. The stress–strain properties of stainless steel were different from those of carbon steel [18,19,20]. There was no yield plateau in the stainless steel curve [21]. The elastic segment of stainless steel was smaller than that of carbon steel [22]. Considering the above differences in stress–strain properties, the stainless steel component performed different resistant properties, compared with carbon steel components [22]. Many researchers paid attention to the aforementioned issue [15,23,24,25,26]. With increments in the development of stainless steel, the new S600E sorbite stainless steel (SS) realized quantitative production. Compared with the commonly used S30408 stainless steel, the S600E sorbite SS performed similar corrosion resistance. The strength properties of the S600E sorbite SS were better than those of S30408 stainless steel. Furthermore, the contents of precious metallic elements, such as Ni and Cr, of the S600E sorbite SS were lower than those of traditional stainless steel. Hence, the S600E sorbite SS’s price had an obvious competitive advantage [13]. Some researchers on the S600E sorbite SS stated the advantage for engineering applications [27,28]. However, the research on the S600E sorbite SS component was very limited. Considering outstanding corrosion resistance and strength properties, the innovative usage of S600E sorbite SS could bring beneficial effects on the durability and resistance of the component, especially when the plate thickness was relatively small.

**Figure 2:**
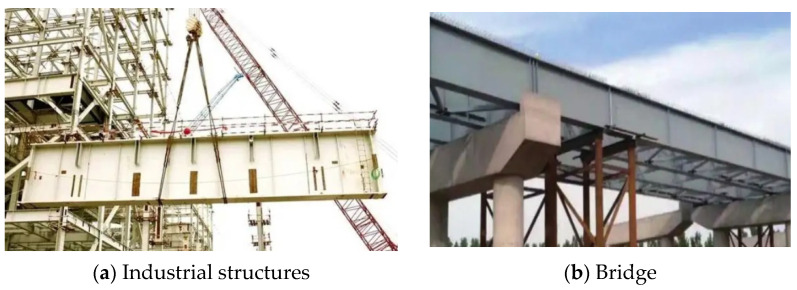
Plate girder engineering application.

The plate girder is an important component in bridge and industrial structures, and has an excellent load-resistant capacity. The plate girder is usually composed of flange, web, and transverse stiffener (Figure 2). To improve the resistant properties and reduce steel consumption, the thickness of the plate girder web is usually small. When the shear and patch loading were applied on the plate girder with a thin web, the post-buckling capacity occurred in the plate girder, which performed beneficial effects on resistant properties. Because of the post-buckling capacity, the ultimate resistance of the plate girder was not dependent on the buckling resistance but the mechanical properties of structural steel, which enabled the full utilization of material properties. Many researchers performed investigations on resistant properties of plate girders developing post-buckling capacity. Xiao et al. [29,30] studied the ultimate shear resistance of Q690 high-strength steel plate girder numerically. The results stated that with the increment in strength properties of structural steel, the ultimate shear resistance of the plate girder increased gradually. Nonlinear properties of structural steel also influenced the resistance properties of the plate girder. Hua et al. [7] revealed the residual resistance of Q690 high-strength steel plate girder under shear. The effects from the exposure temperature were reflected in the changes of stress–strain properties of structural steel, which were introduced in the plate girder numerical model. Numerical results indicated that the existence of yield plateau reduced the ultimate shear resistance of the plate girder. Saliba and Gardner [31] conducted experimental and numerical investigations on a lean duplex stainless steel plate girder under shear, and validated the equations in EN 1993-1-4+A1 [32]. It is worth noting that Xue et al. [28] clarified the ultimate shear resistance of the S600E sorbite SS plate girder. However, the distance between flange plastic hinges was determined on the failure mode of the plate girder model, which was not clear enough. The above current investigations on plate girders [33,34,35,36] stated that different mechanical properties resulted in differences in the resistant properties of the plate girder with a post-buckling capacity. It should be noted that the design methods in EN 1993-1-5 [37] came from investigations on the carbon steel plate girder [38], where the rotated stress field theory was proposed. Then, Olsson [39] and Saliba et al. [40] considered the nonlinear characteristics of stainless steel and proposed the modified design equations based on EN 1993-1-5 [37], which were included in EN 1993-1-4+A1 [32]. There were differences in the stress–strain properties between S600E sorbite SS and traditional stainless steel [27,28], which might result in differences in plate girder resistant properties. The current design equations might be inappropriate to predict the ultimate resistance of the S600E sorbite SS plate girder under shear, which limits the engineering application of the S600E sorbite SS. The research on the distance between flange plastic hinges was very limited, which was important for the resistance from the flange. A new method was proposed in this study to clarify the resistance from the flange. As a common research method, a finite element analysis has been widely used to study the bearing capacity and deformation of various engineering structures [41,42,43,44,45]. In this study, the series finite element analyses on the S600E sorbite SS plate girder under shear were conducted to clarify the advantage of the usage of the S600E sorbite SS. The effects of the strain hardening properties of structural steel on the resistant properties of the plate girder were discussed. After verification, the design equations in EN 1993-1-4+A1 [32] could not directly predict the resistance from the flange of S600E sorbite SS plate girders. Based on the parameter study, a new method was suggested to determine the resistance from flange. Then, the design equations in EN 1993-1-4+A1 [32] were modified to predict the resistance from the flange of the S600E sorbite SS plate girders accurately. The research in this study clarified the resistant properties of the S600E sorbite SS plate girder under shear. The design equations were suggested to quantify the ultimate shear resistance of the S600E sorbite SS plate girder, which performed the engineering significance.

## 2. Finite Element Analysis

### 2.1. Mechanical Property

**Figure 3:**
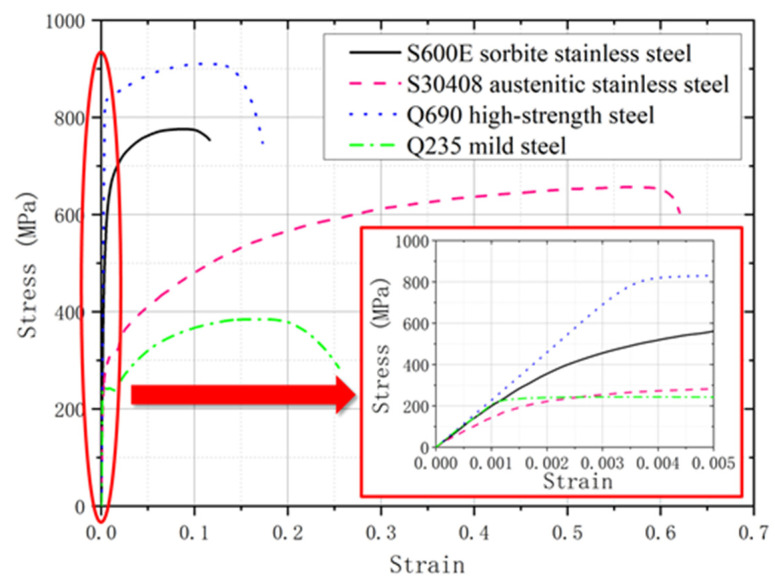
Comparison in stress–strain curves between stainless steel and carbon steel [27,29].

To clarify the mechanical property of the S600E sorbite SS comprehensively, the comparison in stress–strain curves among S600E sorbite SS, S30408 austenitic stainless steel, Q235 mild steel, and Q690 high–strength steel was performed (Figure 3). The experimental results of S30408 austenitic stainless steel were obtained from [27]. The experimental results of Q235 mild steel and Q690 high-strength steel were obtained from [29]. It is clear to see that the Q690 high-strength steel performed the highest strength properties. There were clear elastic segments in the stress–strain curves of Q235 mild steel and Q690 high-strength steel. For the S600E sorbite SS and S30408 austenitic stainless steel, when the stress level was relatively low, there was plastic strain in the stress–strain curve. This phenomenon might be caused by differences in chemical composition (Table 1). It is worth noting that there was no yield plateau in the stress–strain curves of Q690 high-strength steel, S600E sorbite SS, and S30408 austenitic stainless steel [46,47]. It is worth noting that for the stress–strain curve without a clear yield plateau, the yield strength *f_y_* was the nominal stress corresponding to 0.2% proof stress [48,49,50]. The ductile properties of S600E sorbite SS were lower than those of Q690 high-strength steel. Because of the above differences in the stress–strain properties of the different structural steels, the current design equations for the traditional structural steel plate girder might not be applicable to the S600E sorbite SS plate girder. The true stress–strain properties were obtained from the engineering stress–strain properties. Considering that the necking phenomenon could be simulated by a numerical model appropriately, the true stress–strain properties should be introduced in a numerical model to achieve the better accuracy. It is worth noting that in Section 2.5, the elastic-perfectly plastic stress–strain curve was used to clarify the effects of the strain hardening effect on the resistant properties of the S600E sorbite SS plate girder. Except the discussion in Section 2.5, the true stress–strain curve of S600E sorbite SS was considered to be the constitutive model of the numerical analysis.

### 2.2. Numerical Method

**Figure 4:**
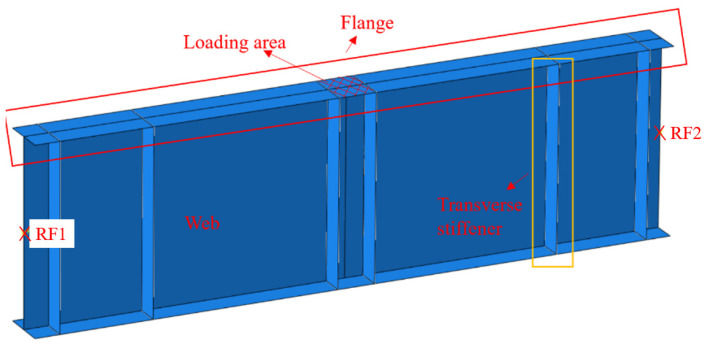
Structural form of plate girder model.

**Figure 5:**
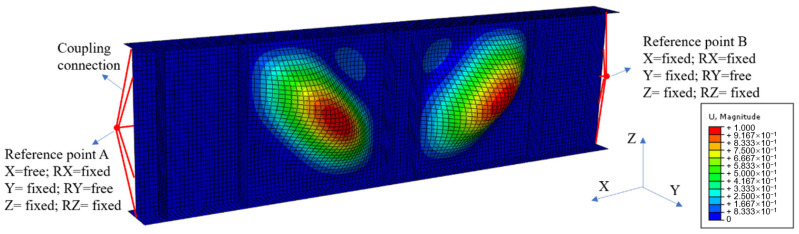
First buckling mode of the S600E sorbite SS plate girder.

The numerical analysis was conducted to investigate the ultimate shear resistance of the S600E sorbite SS plate girder. Similar to the numerical analyses in [7,29,30], the shell element was selected to simulate the plate girder, whose plate thickness was relatively small. The S4R element, whose node contained three rotational and three translational degrees-of-freedom, was used to build the flange, transverse stiffener, and web (Figure 4). A tie connection was used among the flange, transverse stiffener, and web. The simply supported boundary condition was achieved through the reference point and coupling connection. The end sections of the plate girder model were connected with reference points A and B, respectively, where the coupling connection was used. Then, the rotational and translational degrees-of-freedom of the node on the end sections of the plate girder model were the same as those of reference points A and B, respectively. The rotational and translational degrees-of-freedom of reference points A and B are shown in Figure 5. For reference point A, translational degree-of-freedom X and rotational degree-of-freedom RY were free and other rotational and translational degrees-of-freedom were fixed. For reference point B, rotational degree-of-freedom RY was free and other rotational and translational degrees-of-freedom were fixed. A concentrated load was applied on the top flange area between the transverse stiffener, as shown in Figure 4. Out–of–plane constraints were applied on the top flange to protect the plate girder model from lateral–torsional buckling. It is worth noting that the mesh size performed effects on the efficiency and accuracy of the numerical analysis. After a convergence study including different mesh sizes (5 mm, 10 mm, 20 mm, 40 mm, and 80 mm), the 20 mm was selected to be the mesh size for the numerical analysis in this study.

Initial imperfection brought effects on resistant properties of the plate girder, which should be considered in the numerical model. However, the initial imperfection performed significant uncertainty. Hence, a method based on the buckling mode was used to include the effects of initial imperfection, which was widely accepted by many researchers [29,30,31,51,52]. First, the elastic buckling analysis was performed to have the buckling mode of the plate girder. The out-of-plane buckling occurred in web. No clear buckling deformation was observed in the flange and transverse stiffener. The first buckling mode was widely selected to be the initial imperfection form of the plate girders. Then, based on suggestions in the AASHTO Bridge Welding Code [53], *h_w_*/100 was selected to be the amplitude of initial imperfection. The keywords of the numerical model in ABAQUS 6.14 software were modified to introduce the initial imperfection. After that, the ultimate resistant analysis on the S600E sorbite SS plate girder could be performed to have the ultimate resistance.

### 2.3. Numerical Model Validation

**Figure 6:**
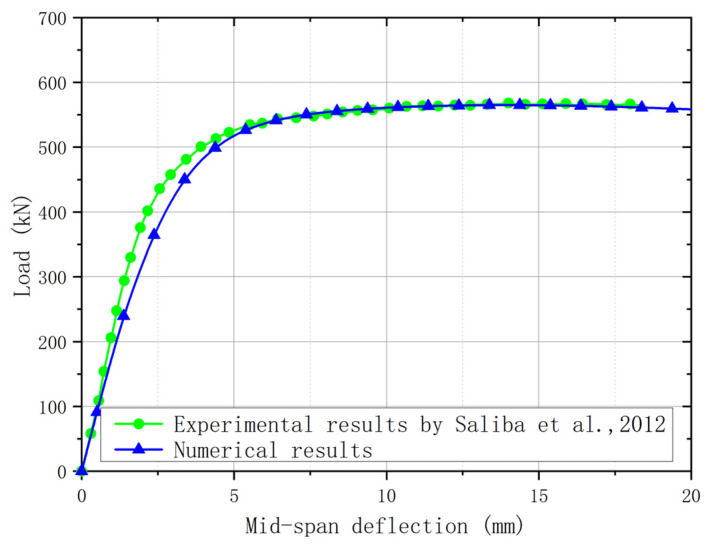
Comparison between experimental [31] and numerical results for plate girder under low-moment high-shear loading.

**Figure 7:**
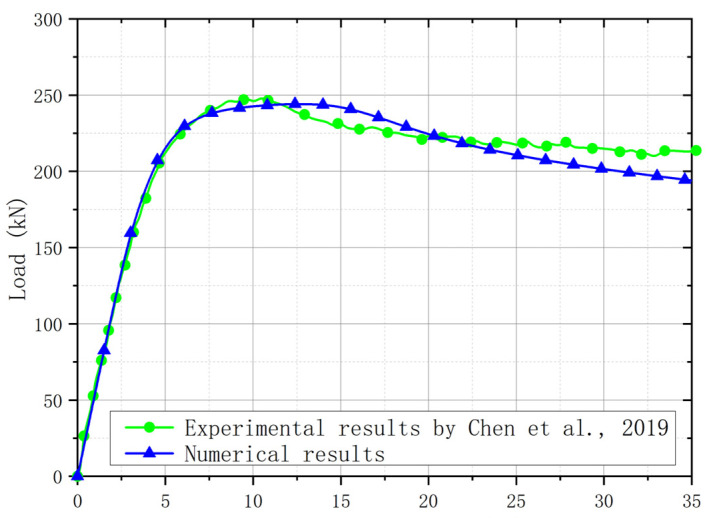
Comparison between experimental [54] and numerical results for the plate girder under high-moment high-shear loading.

**Figure 8:**
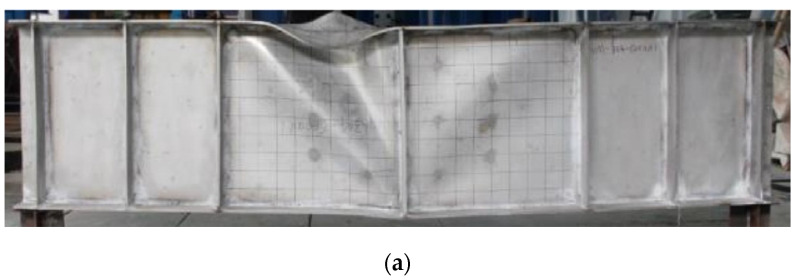

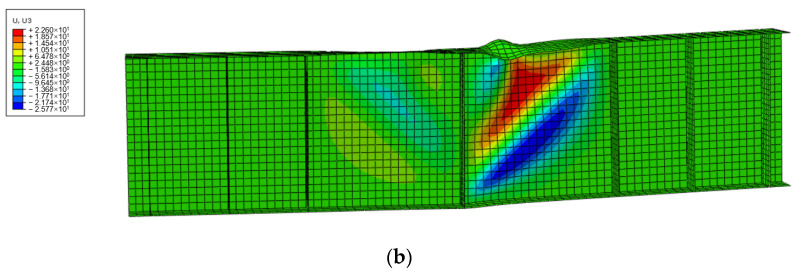
Failure behaviors comparison: (**a**) experiment specimen [54]; (**b**) FE model in this study.

For plate girders with a post-buckling capacity, the resistance from the flange was affected by the bending moment. Hence, it is necessary to validate that the FE model was accurate for plate girders under the low-moment high-shear and high-moment high-shear loading conditions. When the bending moment load was lower than the yield bending moment resistance of the flange, it is believed that the effects of the bending moment load on the shear resistance of the web were ignorable, which was considered to be the low-moment high-shear loading condition. When the bending moment load was higher than the yield bending moment resistance of the flange, it is believed that the bending moment load performed adverse effects on the shear resistance of the web, which was considered to be the high-moment high-shear loading condition. The experiment for the stainless steel plate girders under shear loading conducted by Saliba et al. [31] was chosen for the low-moment high-shear loading condition. The I-600 × 200 × 12 × 4-1 specimen with *h_w_*/*t_w_* = 150 in [31] was also selected to conduct the comparison (Figure 6), which was fabricated with the lean duplex stainless steel. Then, the above comparison stated that the numerical analysis method was appropriate for the stainless steel plate girder under low-moment high-shear loading. For the high-moment high-shear loading, the experimental results in [54] were included. The VM-304-600ad1 specimen with *h_w_*/*t_w_* = 150 was made with austenitic grade EN 1.4301. A comparison between the experimental and numerical results was shown in Figure 7. Furthermore, a comparison between the failure modes for the experiment and numerical model was conducted, as shown in Figure 8. It is worth noting that for the experimental results, the out-of-plane deformation in one web panel was more obvious than that of another web panel. For the numerical results, the out-of-plane deformation was also observed in both web panels. The numerical results agreed well with the experimental results. The flange local buckling and web tension filed were simulated precisely. Hence, it is clear to see that the numerical analysis method could be used to predict the resistant properties of the stainless steel plate girder under the high-moment high-shear loading accurately. Nevertheless, the above numerical analysis method was used by many researchers [29,30,52,55]. Based on the comprehensive validation, the proposed numerical analysis method could be used to clarify the resistance from flange for the S600E sorbite SS plate girder developing the post-buckling capacity.

### 2.4. Specimen Dimensions

To investigate the resistance from the flange for the S600E sorbite SS plate girders with post-buckling capacity, the contribution from the web and the flange should be separated from each other, through *V_bf,Rd_* = *V_b,Rd_* − *V_bw,Rd_*. The *V_b,Rd_* denoted the ultimate resistance of the plate girder. The *V_bf,Rd_* and *V_bw,Rd_* denoted the resistance from the flange and web, respectively. Based on the design equations in EN 1993-1-4: 2006+A1 and the study conducted in [29,56], the *V_bw,Rd_* was affected by the dimensions of the web and flange only, when the failure mode was the flange plastic hinge mode. It is believed that when the section dimension of the plate girder was controlled to be the same, the resistance from the web *V_bw,Rd_* remained unchanged. Hence, for the finite element analysis (FEA), the section dimensions of plate girders in the same group were controlled to be the same, which stated that the resistance from the web *V_bw,Rd_* remained unchanged for plate girders in the same group.

**Figure 9:**
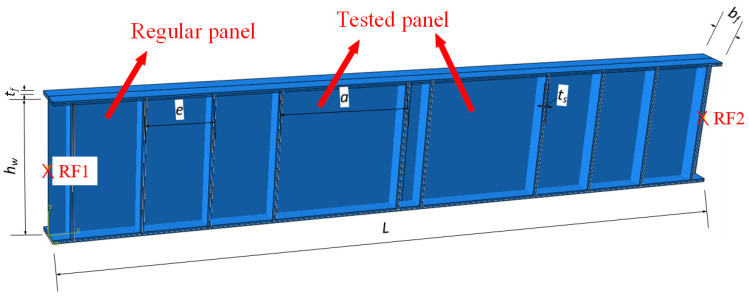
Structure form of plate girder model.

**Figure 10:**
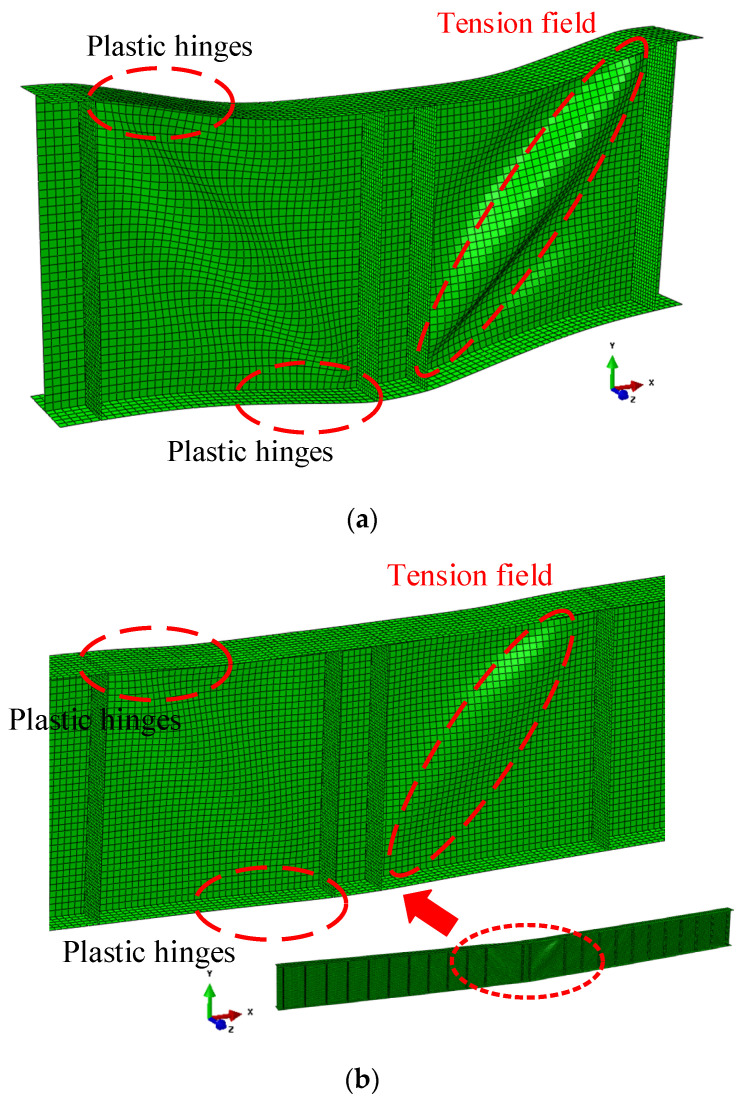
Failure modes of S600E sorbite SS plate girders: (**a**) P1-1-150-40; (**b**) P11-1-150-40.

For the FEA conducted in this study, the numerical model consisted of the tested panels, the regular panels, the flanges, and the transverse stiffeners, as shown in Figure 9. For the tested panels, they were symmetrically distributed at the mid-span area with the maximum bending moment and shear loading. For the regular panels, they were symmetrically distributed on both sides of the tested panels. The number of the regular panel was controlled to perform the different ratios of the bending moment and shear loading, as shown in Table 2. Then, the different ratios of *δ* = *M_Ed_*/*M_f,k_* could be obtained, where *M_f,k_* was the moment of resistance of the cross-section, consisting of the effective area of the flanges only. For all specimens, the thickness of the transverse stiffener was 20 mm. The transverse stiffener was rigid enough to keep the nodal line when the ultimate resistance was exceeded. Consequently, no transverse stiffener buckling failure mode was observed. The flange plastic hinge mode was the only failure mode of specimens, as shown in Figure 10. It can be concluded that the post-buckling capacity of the S600E sorbite SS plate girder was fully developed [29].

**Figure 11:**
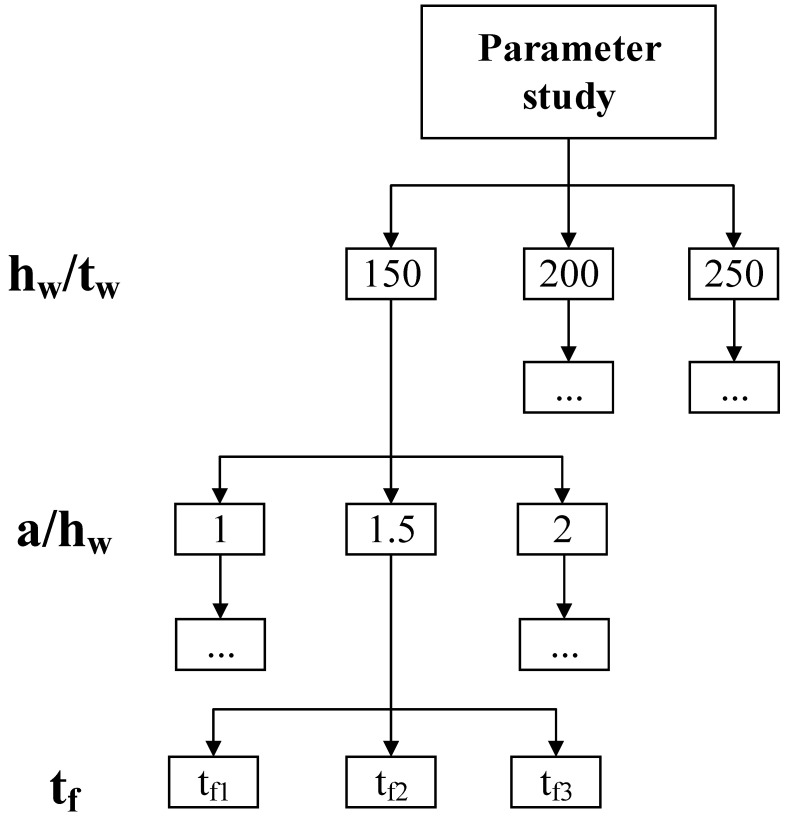
Schematic representation of numerical parameter study.

In this study, the parametric research for the S600E sorbite SS plate girders with post-buckling capacity was conducted, in which the different flange thicknesses, depth ratios *h_w_/t_w_*, and aspect ratios *a*/*h_w_* were analyzed (see Figure 11). The height of the web was 750 mm. The different depth ratios *h_w_/t_w_* were obtained by changing the thickness of the web. P1-1-150-40 indicated that *a*/*h_w_* =1, *h_w_*/*t_w_* = 150, and *t_f_* = 40 mm. In terms of the development of the post-buckling capacity and engineering practicability, the *h_w_*/*t_w_* =150/200/250 and aspect ratio *a*/*h_w_* = 1/1.5/2 were designed. The width of the flange was 200 mm. To investigate the resistance from flange and avoid the flange buckling mode, different flange thicknesses were included in the FEA, as shown in Table 3.

### 2.5. Effect of Strain Hardening

**Figure 12:**
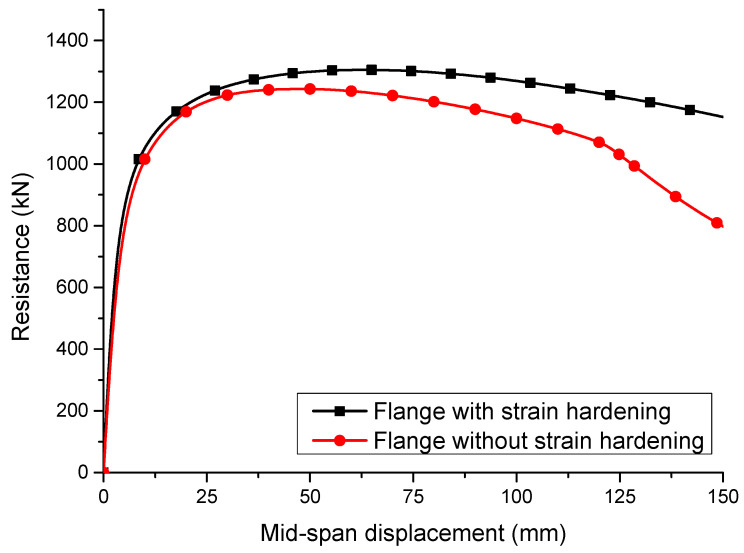
Effect of strain hardening on the resistance.

The P1-1-150 plate girder with *t_f_* = 30 mm in Table 2 was selected to discuss the effect of strain hardening on the resistance from the flange for the S600E sorbite SS plate girders considering the post-buckling capacity. For the web and the transverse stiffener, the material model included the strain hardening effect. For the flange, the material models with and without the strain hardening effect were used to perform the FEA, separately. For the numerical model without the strain hardening effect, the elastic-perfectly plastic stress–strain curve was used in the flange. For the numerical model with the strain hardening effect, the true stress–strain curve was used in the flange. A comparison between these two FEA results was conducted, as shown in Figure 12. It is clear to see that the material strain hardening in the flange improved the resistance of the S600E sorbite SS plate girders considering the post-buckling capacity, while the flange resistance was not fully used to resist the bending moment. Given that the strain hardening properties of the S600E sorbite SS were different from those of other types of stainless steel, it is significant to study the resistance from the flange for the S600E sorbite SS plate girders considering the effect of strain hardening properties. It should be noted that for all numerical analyses conducted in this study, the strain hardening properties of the S600E sorbite SS were fully considered.

## 3. Current Design Method

Design methods in EN 1993-1-5 [37] for the carbon steel plate girder and EN 1993-1-4+A1 [32] for the stainless steel plate girder are introduced in this section. It should be noted that the design methods in EN 1993-1-4+A1 [32] were modified from EN 1993-1-5 [37], where the mechanical properties of stainless steel were considered. The design equations in EN 1993-1-5 [37] came from the rotated stress field theory proposed by Höglund [38], which stated the variation trends of compressive stress in the web. Ultimate resistance was determined by Equation (1). The *V_bw.Rd_* and *V_bf.Rd_* were resistance from the web and flange, respectively. Strain hardening properties of stainless steel were considered through the *η* = 1.20. *V_bw.Rd_* was calculated through Equation (2). It is worth noting that when flange resistance was not completely utilized in resisting the bending moment (*M_Ed_* < *M_f,Rd_*), the *V_bf.Rd_* could be estimated by Equation (3). For the mild steel plate girder, the *c* was determined by Equation (4) in Part 1-4 of EN 1993-1-5 [37]. For the stainless steel plate girder, Equation (5) in EN 1993-1-4+A1 [32] was suggested, which was based on the experimental results in [39].


(1) $$V_{b,Rd}=V_{bw,Rd}+V_{bf,Rd}\leq \frac{\eta f_{yw}A_{w}}{{\sqrt{3}}_{\gamma_{M1}}}$$

(2) $$V_{bw,Rd}=\frac{\chi_{w}f_{yw}A_{w}}{{\sqrt{3}}_{\gamma_{M1}}}$$

(3) $$V_{bf,Rd}=\frac{b_{f}t_{f}^{2}f_{yf}}{c\gamma_{M1}}\left(1-{\left(\frac{M_{Ed}}{M_{f,Rd}}\right)}^{2}\right)\;M_{Ed}<M_{f,Rd}$$

(4) $$c=a\left[0.25+\frac{1.6b_{f}t_{f}^{2}f_{yf}}{t_{w}h_{w}^{2}f_{yw}}\right]$$

(5) $$c=a\left[0.17+\frac{3.5b_{f}t_{f}^{2}f_{yf}}{t_{w}h_{w}^{2}f_{yw}}\right]\;c/a\leq 0.65$$

## 4. FEA Results Discussion

### 4.1. Failure Behavior

**Figure 13:**
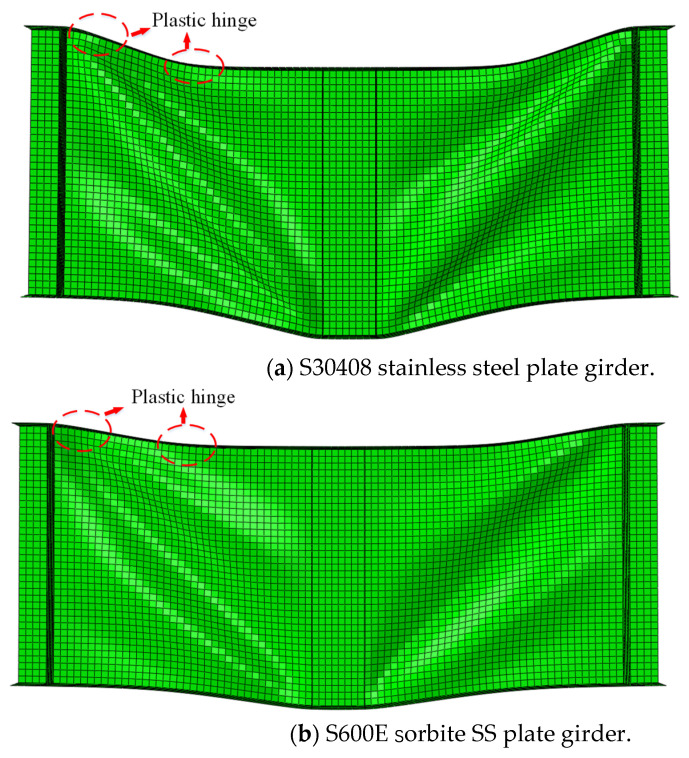
Failure behavior of the S30408 and S600E stainless steel plate girders.

The stress–strain properties of the S30408 stainless steel and S600E sorbite SS in Section 2.1 were used in the P1-1-150 numerical model. After the numerical analysis, the failure behavior corresponding to the ultimate resistance of the S600E sorbite SS plate girder was similar with that of the S30408 stainless steel plate girder (see Figure 13). However, the deformation for the S30408 stainless steel plate girder was more obvious than that of the S600E sorbite SS plate girder. Hence, under the same deformation limit, the S600E sorbite SS plate girder could achieve better resistant performance, compared with the S30408 stainless steel plate girder.

**Figure 14:**
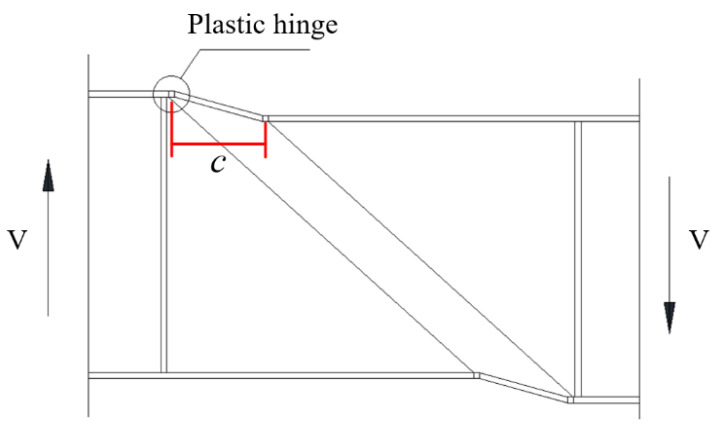
Tension field mechanism for post-buckling stage.

**Figure 15:**
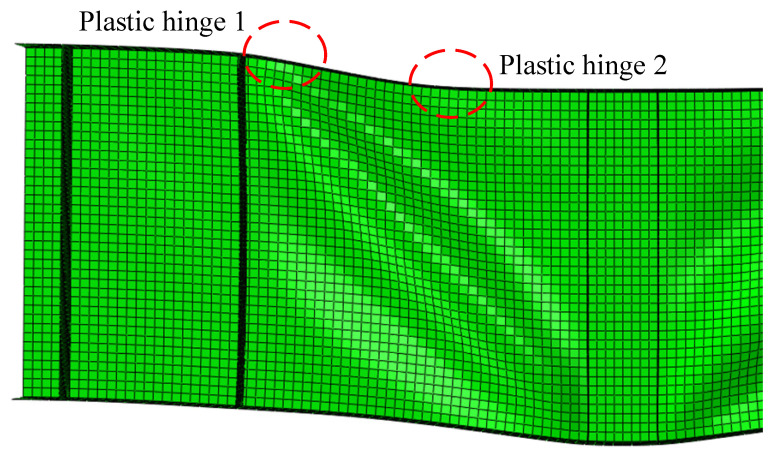
Distribution range of flange plastic hinges.

In the rotated stress field theory proposed by Höglund [38] which was the theoretical basis for EN 1993–1–5 [37], the *c* was the distance between plastic hinges, as shown in Figure 14. For the numerical study considering the material and geometrical nonlinearity, the deformation behavior of the S600E sorbite SS plate girder at the post-buckling stage is shown in Figure 15. It is clear to see that there was a certain distribution range of the flange plastic hinge. It is difficult to determine the distance between the plastic hinges accurately based merely on the failure behavior.

**Figure 16:**
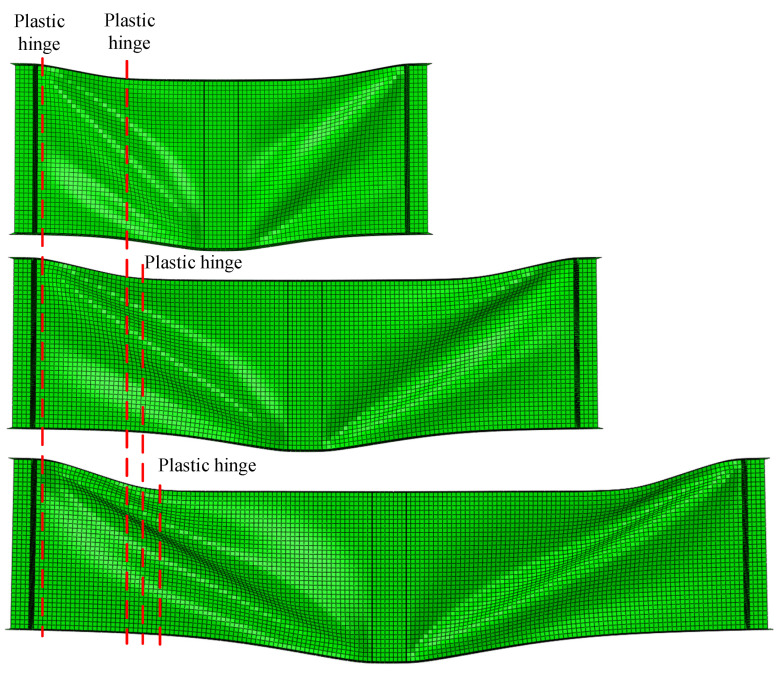
Failure behaviors of the P1 plate girders for 1-150-35, 1.5-150-35, and 2-150-35.

After the FEA, the failure behavior of the P1 plate girder in groups of 1-150-35, 1.5-150-35, and 2-150-35 were obtained, as shown in Figure 16. The 1-150-35 denoted that the *a*/*h_w_* = 1, *h_w_*/*t_w_* =1 50, and *t_f_* = 35 mm. The failure mode was the typical flange plastic hinge mode. For the design equations in EN 1993-1-4+A1, Equation (5) was suggested to determine the *c* for the stainless steel plate girder. However, given Equation (5), the ratio *c*/*a* was not affected by *a*, which was the distance between the transverse stiffeners. As mentioned before, there was a certain distribution range of the flange plastic hinge. It was difficult to determine the distance between the plastic hinges accurately based merely on the failure behavior. However, it is still clear to see that the ratio *c*/*a* was affected by the *a*/*h_w_*. Therefore, it might be inappropriate to use the design equation in EN 1993-1-4+A1 to predict the *c* of S600E sorbite SS plate girders directly. Further verification of the design equation in EN 1993-1-4+A1 is presented in Section 4.3.

### 4.2. Separation between V_bw,Rd_ and V_bf,Rd_

**Figure 17:**
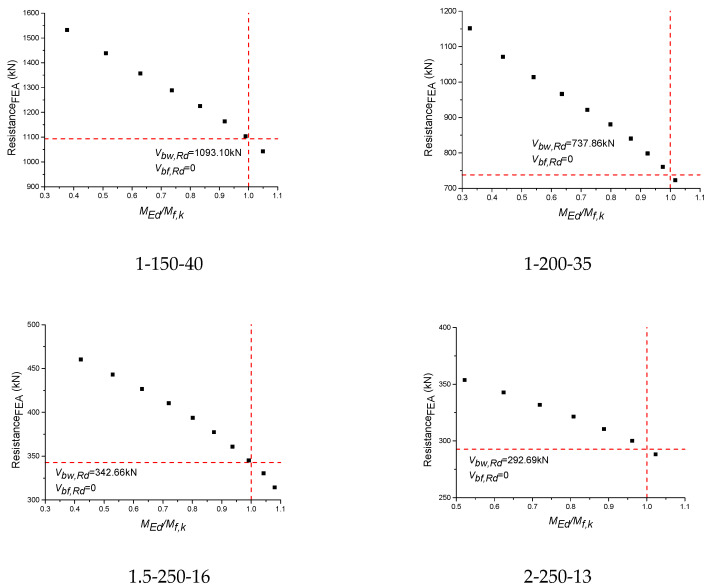
FEA results of S600E sorbite SS plate girders under the different bending moments and shear loadings.

A new method to determine the *c* was proposed in this study based on the theoretical basis of EN 1993-1-4+A1 design equations. After the FEA, the resistances for S600E sorbite SS plate girders under the different combined loading were obtained, as shown in Figure 17. The red lines in Figure 17 were guidelines, which were used to determine the condition of *V_bf.Rd_* = 0. The point in Figure 17 were ultimate resistance from numerical results. The 1-150-40 denoted that the *a*/*h_w_* = 1, *h_w_*/*t_w_* = 150, and *t_f_* = 40 mm. It is clear to see that with increases in the *M_Ed_*/*M_f,k_*, the resistance decreased correspondingly. Considering the theoretical basis of EN 1993-1-4+A1, when *M_Ed_* = *M_f,k_*, it is believed that *V_bf,Rd_* = 0. Then, the resistance of the plate girder was contributed by *V_bw,Rd_* only. For *M_Ed_* = *M_f,k_*, a linear interpolation was conducted based on the FEA results. The variable spacing was small, so the results obtained by linear interpolation were considered to be accurate. After that, the *V_bf.Rd_* could be calculated through *V_bf.Rd_* = *V_b.Rd_* − *V_bw.Rd_*. To clarify the above method clearly, the results for the 1.5-150-40 plate girders were processed as an example, as shown in Table 4. Numerical results stated that the turning point was between P9-1.5-150-40 and P10-1.5-150-40. Then, the *V_bw,Rd_* was obtained through linear interpolation. After that, *V_bf.Rd_* was obtained, where the *c* could be obtained through Equation (3).

### 4.3. Verification of EN 1993-1-4+A1 Formula

**Figure 18:**
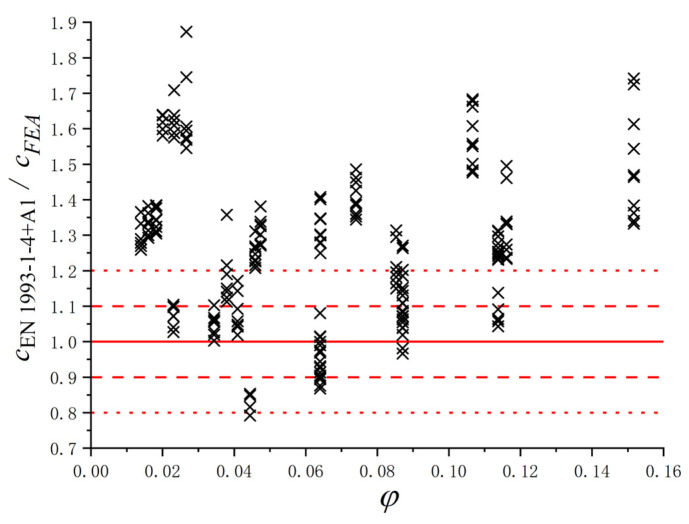
Comparison between the results from numerical analysis and the EN 1993-1-4+A1 design equations for S600E sorbite SS plate girders with post-buckling capacity.

Based on the parametric study, the results of *c* for the S600E sorbite SS plate girders with different geometric dimensions were obtained. To verify whether the design equation in EN 1993-1-4+A1 [32] could predict the *c* accurately, the results of FEA and EN 1993-1-4+A1 design equations were compared (see Figure 18). The red lines in Figure 18 were guidelines, which were used to clarify the difference in *c* between the numerical results and results from design equation. The points were c_EN 1993-1-4+A1_/c_FEA_ with different *φ*. The *φ* in Figure 18 could be calculated by Equation (6). The results indicated that there were noteworthy errors between the FEA results and the results from Equation (5) in EN 1993-1-4+A1, especially when the *φ* was relatively large and small. Therefore, the design equation in EN 1993-1-4+A1 [32] could not be directly used to predict the distance between plastic hinges on the flange. Then, considering that the effects of *c* on the resistance from flange were significant, the *V_bf.Rd_* of the S600E sorbite SS plate girders considering the post-buckling capacity could not be predicted precisely by the EN 1993-1-4+A1 design equations. To investigate the reason for the aforementioned error and propose the appropriate predictive equations, a parameter study was conducted in Section 4.4 and Section 4.5, which considered the *h_w_*/*t_w_*, *a*/*h_w_*, and *M_Ed_*/*M_f,k_*.

(6) $$\varphi =b_{f}t_{f}^{2}f_{yf}/t_{w}h_{w}^{2}f_{yw}$$

### 4.4. Influence of the Depth Ratio h_w_/t_w_ and Aspect Ratio a/h_w_

**Figure 19:**
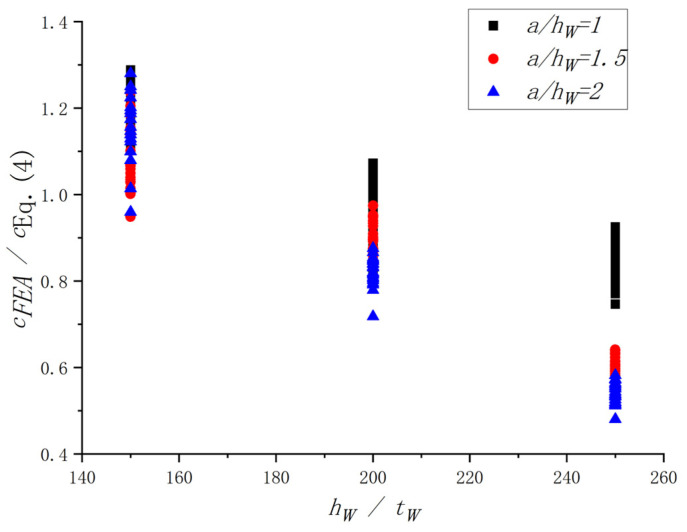
Influence of the *h_w_*/*t_w_* on the *c_FEA_*/*c_Eq. (4)_*.

**Figure 20:**
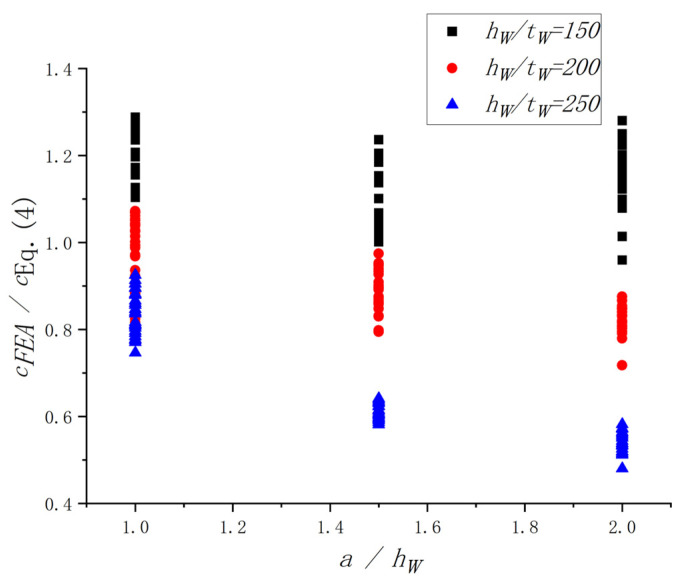
Influence of the *a*/*h_w_* on the *c_FEA_*/*c_Eq. (4)_.*

The original equation proposed by Höglund [38], Equation (4), was selected in this study to investigate the influence of the *h_w_*/*t_w_*. The results of FEA and Equation (4) were compared (see Figure 19). It is easy to see that the *h_w_*/*t_w_* had influenced the *c_FEA_*/*c_Eq. (4)_*. With increases in the *h_w_*/*t_w_*, the *c_FEA_*/*c_Eq. (4)_* decreased correspondingly. A similar comparison was conducted to investigate the influence of the *a*/*h_w_*, as shown in Figure 20. It turns out that the effect of the *a*/*h_w_* on the *c_FEA_*/*c_Eq. (4)_* could not be ignored. Therefore, to predict the *c* accurately, the influence of the *h_w_*/*t_w_* and *a*/*h_w_* should be considered. It is worth noting that the above values of *c_FEA_* were obtained through the method in Section 4.2.

### 4.5. Influence of Ratio M_Ed_/M_f,k_

**Figure 21:**
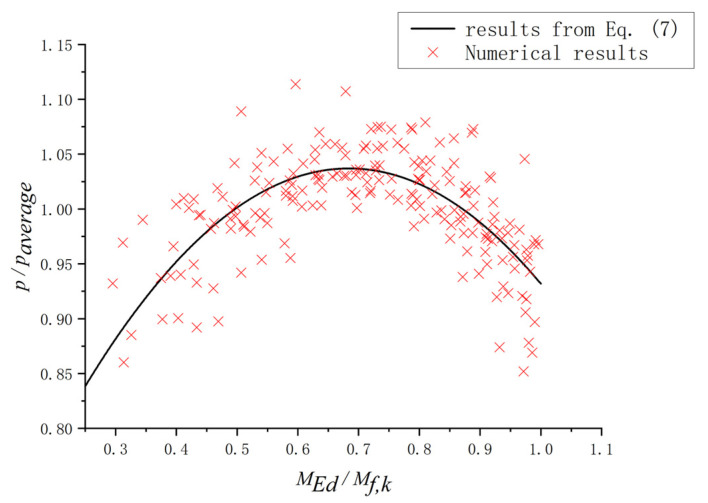
Comparison between numerical results and results from Equation (7).

After the numerical analysis, it turned out that for the plate girders with the same section dimensions, the *c* changed with different bending moments and shear loadings. However, for EN 1993-1-4+A1 [32], the *c* was not influenced by the *γ* = *M_Ed_*/*M_f,k_*, as shown in Equation (5). The influence of the *γ* on the *c* should be included to predict the *c* more accurately. To study the effect of the *γ* on the *p* = *c*/*a*, *α* = *p*/*p_average_* was selected to perform a non-dimensional treatment, where *p_average_* was the mean value of the *p* for the plate girders in the same group. For the plate girders in the same group, the *h_w_*/*t_w_*, *a*/*h_w_*, and *t_f_* remained unchanged. Based on the parametric study conducted in this paper, the ratios *p*/*p_average_* for plate girders with different ratios *M_Ed_*/*M_f,k_* are shown in Figure 21. The results indicated that there was a relationship between the *α* and *γ*. Then, a numerical fitting was performed to clarify the aforementioned relationship between the *α* and *γ*. Equation (7) was proposed to quantify the influence of the *M_Ed_*/*M_f,k_*.

(7) $$\alpha =-1.054\gamma^{2}+1.442\gamma +0.544$$

## 5. Modification for Design Equation in EN 1993-1-4+A1

**Figure 22:**
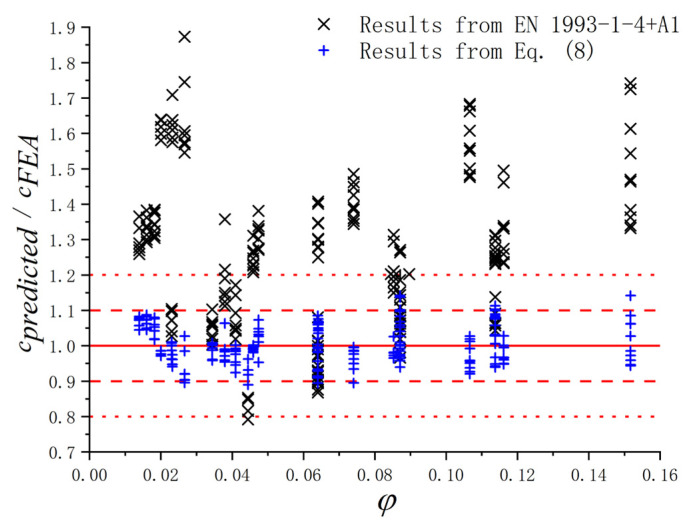
Comparison between the results from FEA, the EN 1993-1-4+A1 design equation for stainless steel plate girders, and the new modified Equation (8).

To predict the resistance from the flange accurately, the design equation in EN 1993-1-4+A1 for the *c* needed to be modified. Considering that the design equations in EN 1993-1-5 [37] and EN 1993-1-4+A1 [32] were all based on the rotated stress field theory proposed by Höglund [38], a new correction factor *ρ* for the original Equation (4) proposed by Höglund [38] was suggested in this study, as shown in Equation (8). Based on the parametric study conducted in this paper, the values of the *c* for the S600E sorbite SS plate girders with different *h_w_*/*t_w_*, *a*/*h_w_*, *t_f_*, and *M_Ed_*/*M_f,k_* were obtained. After the numerical fitting, the correction factor *ρ* could be estimated utilizing Equation (9), which included the influence of the *h_w_*/*t_w_*, *a*/*h_w_*, and *M_Ed_*/*M_f,k_*. It is worth noting that the effects of *M_Ed_*/*M_f,k_* could be reflected by *α*. To verify the accuracy, the FEA results, the results from Equation (5) in EN 1993-1-4+A1, and the new modified Equation (8) were compared, as shown in Figure 22. It could be found that the error associated with Equation (8) was basically controlled within 10%, which was obviously smaller than that of Equation (5). For Equation (8), the average ratio *c_Eq. (8)_*/*c_FEA_* = 1.001 and the coefficient of variance *C_v,Eq. (8)_* = 0.055. For Equation (5) in EN 1993-1-4+A1, *c_Eq. (5)_/c_FEA_* = 1.262 and *C_v,Eq. (5__)_* = 0.217. Consequently, the new modified Equation (8) could predict the resistance from the flange more accurately.

(8) $$c=a\left(0.25+\frac{1.6b_{f}t_{f}^{2}f_{yf}}{t_{w}h_{w}^{2}f_{yw}}\right)\rho$$

(9) $$\rho =\left(1.333+0.3465\frac{a}{h_{w}}-0.0009074\frac{h_{w}}{t_{w}}-0.002583\frac{a}{t_{w}}\right)\alpha$$

## 6. Conclusions

In the study, the S600E sorbite SS was introduced in the plate girder to increase its resistant properties and durability. The resistance from the flange of the S600E sorbite SS plate girders developing the post-buckling capacity was studied based on a numerical analysis. The main conclusions are as follows:

1. It is difficult to determine the distance between the plastic hinges accurately based merely on the failure behavior. Therefore, considering the theoretical basis of EN 1993-1-4+A1, a new method to determine the *c* was proposed to avoid the aforementioned error.

2. The failure behavior for the S600E sorbite SS plate girders with different *a*/*h_w_* was obtained. It indicated that with differences in the *a*/*h_w_*, the *c*/*a* changed correspondingly. The aforementioned influence was not included in the design equations in EN 1993-1-4+A1.

3. The results showed that the error between the numerical analysis and the EN 1993-1-4+A1 design equation should not be ignored. The design equation in EN 1993-1-4+A1 could not be directly selected to predict the resistance from the flange for the S600E sorbite SS plate girders with post-buckling capacity.

4. Based on the parametric study, the results indicated that the *h_w_*/*t_w_*, *a*/*h_w_*, and *M_Ed_*/*M_f,k_* had effects on the value of the *c*. After the numerical fitting, the correction factor *ρ* was proposed, which included the influence of the *h_w_*/*t_w_*, *a*/*h_w_*, and *M_Ed_*/*M_f,k_*.

Considering the advantage of the S600E sorbite SS, it could be widely used in structures in corrosive environments. The transverse patch loading was commonly applied on the plate girder, such as wheel load. The resistant properties of the S600E sorbite SS plate girder under patch loading should be investigated.

## Figures and Tables

**Table 1:** Chemical composition of different structural steels (wt. %).

Type	C	Si	Mn	P	S	Cr	Ni	Alt	Mo	Ti	Cu	Nb	V
S600E sorbite stainless steel	0.09	0.47	0.79	0.077	0.004	13.43	1.46	–	–	–	–	–	–
S30408 austenitic stainless steel	≤0.08	≤0.75	≤2.0	≤0.045	≤0.03	18–20	8–10.5	–	–	–	–	–	–
Q235 mild steel	0.16	0.14	0.53	0.031	0.026	0.014	0.014	–	–	–	–	–	–
Q690 high-strength steel	0.148	0.27	1.23	0.016	0.004	0.17	0.01	0.38	0.129	0.017	0.01	0.02	0.001

**Table 2:** Geometric dimensions for S600E sorbite SS plate girders: *a*/*h_w_* = 1, *h_w_*/*t_w_* = 150.

Number	*L*/mm	*h_w_*/mm	*b_f_*/mm	*a/h_w_*	N × *e*/mm	*t_s_*/mm
P1-1-150	1850	750	200	1	0 × 375	20
P2-1-150	2600	750	200	1	2 × 375	20
P3-1-150	3350	750	200	1	4 × 375	20
P4-1-150	4100	750	200	1	6 × 375	20
P5-1-150	4850	750	200	1	8 × 375	20
P6-1-150	5600	750	200	1	10 × 375	20
P7-1-150	6350	750	200	1	12 × 375	20
P8-1-150	7100	750	200	1	14 × 375	20
P9-1-150	7850	750	200	1	16 × 375	20
P10-1-150	8600	750	200	1	18 × 375	20
P11-1-150	9350	750	200	1	20 × 375	20

**Table 3:** Different *t_f_* for S600E sorbite SS plate girders.

Number	*t_f_*/mm
P1-1-150~P11-1-150	30/35/40
P1-1.5-150~P10-1.5-150	30/35/40
P1-2-150~P9-2-150	25/30/35
P1-1-200~P11-1-200	30/35/40
P1-1.5-200~P10-1.5-200	22/24/26
P1-2-200~P9-2-200	18/20/22
P1-1-250~P11-1-250	20/25/30
P1-1.5-250~P10-1.5-250	14/15/16
P1-2-250~P9-2-250	13/14/15

**Table 4:** Numerical results for S600E sorbite SS plate girders: 1.5-150-40.

Number	*V_b.Rd_*/kN	*M_Ed_*/*M_f,k_*	*V_bf.Rd_*/kN	*c_FEA_*/mm	*c_Eq. (5)_*/mm
P1-1.5-150-40	1114.51	0.395	303.10	486.90	639.25
P2-1.5-150-40	1074.25	0.498	262.84	500.67	639.25
P3-1.5-150-40	1034.43	0.591	223.02	509.91	639.25
P4-1.5-150-40	994.15	0.676	182.74	519.29	639.25
P5-1.5-150-40	956.73	0.755	145.32	517.98	639.25
P6-1.5-150-40	919.68	0.825	108.26	515.00	639.25
P7-1.5-150-40	883.86	0.889	72.45	505.46	639.25
P8-1.5-150-40	848.72	0.946	37.31	493.06	639.25
P9-1.5-150-40	814.29	0.996	2.88	487.83	639.25
P10-1.5-150-40	781.90	1.041	-	-	-
